# The 100 Top-Cited Studies About Pain and Depression

**DOI:** 10.3389/fpsyg.2019.03072

**Published:** 2020-02-11

**Authors:** Liang Du, Shanxia Luo, Guina Liu, Hao Wang, Lingli Zheng, Yonggang Zhang

**Affiliations:** ^1^Department of Periodical Press and National Clinical Research Center for Geriatrics, West China Hospital, Sichuan University, Chengdu, China; ^2^Chinese Evidence-Based Medicine Center, West China Hospital, Sichuan University, Chengdu, China; ^3^Mental Health Center, West China Hospital, Sichuan University, Chengdu, China; ^4^West China School of Medicine, Sichuan University, Chengdu, China

**Keywords:** pain, depression, top-cited, bibliometric review, citation, citation analysis

## Abstract

With the estimated high prevalence in the population, the two symptoms of pain and depression threaten the well-being of millions worldwide. Researches of the two symptoms increased year by year. Top-cited studies will help to understand the achievement and guide researchers toward the direction of the research field. However, it is unclear for researches in the field of pain and depression. In this paper, we reviewed the bibliometric characteristics of the top-cited papers about pain and depression. We will review the evidence of authorship, country of origin, institution, journal, study type, and publication year for the 100 top-cited studies on pain and depression based on the Web of Science Core collection. We also highlight studies with the highest cited times. Our study concluded that pain and depression were correlated, which may share common biological pathways.

## Introduction

With the estimated prevalence in the adult population ranges from 5 to 60% (Gureje et al., 1998; Blyth et al., 2001; Smith et al., 2001), the pain has become the most common problem worldwide. Likewise, depression, the first cause of disability before 2030 forecasted by WHO (2017), prevalence of which in primary care is estimated at 5–10% (Katon and Schulberg, 1992). Current evidence suggests that pain and depression have reciprocal influence (Kroenke et al., 2011) and often co-occur (Kroenke et al., 2010). The prevalence of co-occurrence of pain and depression ranges from 30 to 65% (Arnow et al., 2006; Bair et al., 2008), even higher than the respective prevalence of pain and depression (Bair et al., 2003). Growing number of studies investigated the comorbidity of pain and depression (Campbell et al., 2003; Chopra and Arora, 2014; Li, 2015), yet there is no relevant evidence in the aspect of bibliometrics analysis.

Bibliometric method has been widely used to provide an analysis of books and articles and has been used to assess the impact of research outputs (Blakeman, 2018). Citation analysis is one of the key methods of bibliometric method, aiming at constructing indicators of research performance from a quantitative analysis of scholarly documents (Moed, 2009). The frequency of citation indicates the relative significance in the particular discipline (Iyengar et al., 2009; Kanter, 2009). Analysis of top-cited studies helps understand the current achievement and guide researchers toward the direction of development of the field (Zhang et al., 2019a,b).

Analysis of top-cited articles has been used in different fields, including cancer immunotherapy (Zhang et al., 2019a), cardiology (Shuaib et al., 2015), gastroenterology and hepatology (Azer and Azer, 2016), and urology (Ipekci et al., 2017). However, there is no top-cited analysis of the comorbidity of pain and depression. Intend to bridge this gap, in this study, we performed a bibliometirc review to assess 100 top-cited studies on pain and depressions based on Web of Science Core Collection and discuss the relationship between pain and depression.

## Review Methods

Studies of pain and depression published in journals were identified in the Web of Science Core Collection using the keywords “pain” and “depression” in July 2019. The Web of Science Core Collection database includes peer-reviewed publications indexed from more than 10,000 high impact journals in the world (Brown et al., 2019). All published papers since 1945 were searched in the database, and citation count ranked the results. Two researchers independently selected the 100 top-cited studies about pain and depression. Any article studied the comorbidity of pain and depression were included for further analysis. Articles mentioned pain and depression without investigating the relation between pain and depression were excluded. The disagreements were resolved by discussion. If necessary, the discrepancies were resolved by consulting the third researcher. A data extracted form was pre-defined, including the basic information, such as title, the first author and corresponding author's name, publication year, the number of citations, source of the journal, impact factor of the journal, article type, organization of corresponding author, country of origin based on the corresponding author. Each study was reviewed, and the information was collected. The relationships between the number of citations and journal, publication year, study type, country, institution, and authorship were analyzed. Descriptive statistics were used to present the results. The analyzed data and results could be assessed by contact with the corresponding author of this study. This is a bibliometric review, so ethics is not applicable.

## The Citation Characteristics Of The 100 Top-Cited Studies on Pain and Depression

The 100 top-cited studies are listed in Table 1 in descending order of the number of citations, which were published from 1979 to 2014. The number of citations ranged from 94 to 1,576, and the mean citation number was 191. The most frequently cited study was “Depression and pain comorbidity-a literature review” (Bair et al., 2003). It was published in Archives of Internal Medicine (now JAMA Internal Medicine), the author conducted a literature review to determine the prevalence of pain and depression, they also reviewed the effects of comorbidity on diagnosis, clinical outcomes, and treatment. The second most-cited paper was a review named “Chronic pain-associated depression: antecedent or consequence of chronic pain? a review” (Fishbain et al., 1997), which was published in Clinical Journal of Pain, it was cited by other studies about 618 times. The authors reviewed eighty-three studies and found that depression was more common in chronic pain patients (CPPs) than in healthy controls. The third most cited study was a review named “Chronic pain and depression-dose the evidence support a relationship” (Romano and Turner, 1985). It was published in Psychological Bulletin and been cited 523 times.

**Table 1:** The 100 top-cited studies in pain and depression.

**Ranking**	**Author, year**	**Journal**	**Article type**	**Total citation**	**Publication year**	**The relationship**
1	Bair, 2003	*Arch. Intern. Med*.	Review	1576	2003	Positive correlated
2	Fishbain, 1997	*Clin. J. Pain*.	Review	618	1997	Pain caused depression
3	Romano, 1985	*Psychol. Bull*.	Review	523	1985	Pain caused depression
4	Banks, 1996	*Psychol. Bull*.	Review	455	1996	Pain caused depression
5	Tsang, 2008	*J. Pain*.	Trial	420	2008	Pain caused depression
6	Lin, 2003	*Jama-J. Am. Med. Assoc*.	Trial	380	2003	Positive correlated
7	Geisser, 1997	*Clin. J. Pain*.	Trial	335	1997	Pain caused depression
8	Currie, 2004	*Pain*	Trial	291	2004	Pain caused depression
9	McWilliams, 2004	*Pain*	Trial	289	2004	Pain caused depression
10	VonKorff, 1996	*Brit. J. Psychiatr*.	Trial	271	1996	Pain caused depression
11	Campo, 20040	*Pediatrics*	Trial	265	2004	Pain caused depression
12	Blackburn, 2001	*J. Neuroendocrinol*.	Review	258	2001	Both were related to Hypothalamo-pituitary-adrenal HPA axis
13	Rudy, 1988	*Psychosom. Med*.	Trial	256	2004	No correlation
14	Bair, 2004	*Pain*	Trial	252	1988	Pain caused depression
15	Arnstein, 1999	*Pain*	Trial	251	1999	Pain caused depression
16	Gore, 2005	*J. Pain. Symptom. Manag*	Trial	248	2005	Pain caused depression
17	Arnow, 2006	*Psychosom. Med*.	Trial	246	2006	Depression caused pain
18	Kroenke, 2009	*Jama-J. Am. Med. Assoc*.	Trial	241	2009	Positive correlated
19	Giesecke, 2005	*Arthritis Rheum.-Us*	Trial	238	2005	No correlation
20	Bair, 2008	*Psychosom. Med*	Trial	240	2008	Depression caused pain
21	Vonkorff, 1993	*Pain*	Trial	226	1993	No correlation
22	Edwards, 2011	*Nat. Rev. Rheumatol*.	Review	222	2011	Pain caused depression
23	Kroenke, 2011	*J. Pain*.	Trial	220	2011	Positive correlated
24	Campbell, 2003	*Biol. Psychiatr*.	Trial	219	2003	Pain caused depression
25	Sullivan, 1990	*J. Abnorm. Psychol*.	Trial	214	1990	Pain caused depression
26	Kashikar, 2001	*Clin. J. Pain*.	Trial	211	2001	Pain caused depression
27	Raskin, 2007	*Am. J. Psychiatr*.	Trial	208	2007	Positive correlated
28	Carroll, 2004	*Pain*	Trial	204	2004	Depression caused pain
29	Turk, 1994	*Behav. Res. Ther*.	Trial	203	1994	Pain caused depression
30	Brown, 1989	*J. Consult. Clin. Psych*.	Trial	199	1989	Pain caused depression
31	Brown, 1990	*J. Abnorm. Psychol*.	Review	197	1990	Pain caused depression
32	Chwastiak, 2003	*J. Clin. Epidemiol*.	Trial	196	2003	Pain caused depression
33	Brander, 2007	*Clin. Orthop. Relat. R*	Review	189	2007	Pain caused depression
34	Turk, 1995	*Pain*	Trial	186	1995	Pain caused depression
35	Wolfe, 1999	*Rheumatology*	Review	184	1999	Pain caused depression
36	Geisser, 1994	*Pain*	Trial	179	1994	Positive correlated
37	Turner, 1984	*J. Clin. Psychol*.	Trial	178	1984	Pain caused depression
38	Maletic, 2009	*Front. Biosci*.	Review	175	2009	Both were related to dysregulation of stress/inflammatory pathways
39	Eisenach, 2008	*Pain*	Trial	174	2008	Pain caused depression
40	Sullivan, 2001	*Pain*	Trial	171	2001	Pain caused depression
41	Ward, 1979	*Pain*	Trial	168	1979	Depression caused pain
42	Bigatti, 2008	*Arthritis Rheum. Arthr*.	Trial	168	2008	Pain caused depression
43	Lindsay, 1981	*Psychosomatics*	Trial	167	1981	Positive correlated
44	Kroenke, 2010	*Jama J. Am. Med. Assoc*.	Trial	166	2010	Positive correlated
45	SPIEGEL, 1994	*Cancer*	Trial	164	1994	Pain caused depression
46	Parmelee, 1991	*J. Gerontol*.	Trial	163	1991	Depression caused pain
47	Kim, 2012	*J. Clin. Invest*.	Basic research	158	2012	Both were related to brain indoleamine 2,3-dioxygenase 1
48	Haythornthwaite, 1991	*Pain*	Trial	150	1991	Positive correlated
49	Sullivan, 1992	*Pain*	Review	147	1992	Pain caused depression
50	Wilson, 2002	*Clin. J. Pain*	Trial	145	2002	Depression caused pain
51	Diepenmaat, 2006	*Pediatrics*	Trial	145	2006	Pain caused depression
52	Dworkin, 1991	*Clin. J. Pain*	Review	145	1991	Pain caused depression
53	Walker, 2014	*Pharmacol. Rev*.	Review	143	2014	Both were related to several mechanisms
54	Ciechanowski, 2003	*Pain*	Trial	141	2003	Both were related to attachment theory
55	Mullen, 1987	*J. Rheumatol*.	Trial	141	1987	Both were related to psychoeducation
56	Suhr, 2003	*J. Psychosom. Res*.	Trial	141	2003	Both were related to fibromyalgia
57	Nicassio, 1992	*J. Abnorm. Psychol*.	Trial	140	1992	Pain caused depression
58	Karp, 2005	*J. Clin. Psychiatr*.	Trial	139	2005	Positive correlated
59	Dickens, 2003	*Psychosom. Med*.	Review	139	2003	Depression elevated pain perception threshold
60	Bar, 2005	*Pain*	Trial	139	2005	Depression caused lateralized perception of pain
61	Lepine, 2004	*Hum. Psychopharm. Clin*.	Trial	138	2004	Pain caused depression
62	Miller, 2009	*J. Pain*	Trial	134	2009	Pain caused depression
63	Kelsen, 1995	*J. Clin. Oncol*.	Trial	134	1995	Both were not related to recently diagnosed adenocarcinoma of the pancreas
64	Hassett, 2000	*Arthritis Rheum. Us*	Trial	132	2000	Both were related to fibromyalgia
65	Ciaramella, 2001	*Psycho-Oncology*	Trial	132	2001	Depression caused pain
66	Lautenbacher, 1999	*Psychosom. Med*.	Trial	129	1999	Depression elevated pain perception threshold
67	Elliott, 2003	*Pain Med*.	Trial	128	2003	Pain caused depression
68	So, 2009	*Oncol. Nurs. Forum*.	Trial	126	2009	Positive correlated
69	Cairns, 1996	*Arch. Phys. Med. Rehab*.	Trial	125	1996	Positive correlated
70	Williamson, 1992	*J. Gerontol*.	Trial	122	1992	Positive correlated
71	Hawker, 2011	*Arthritis Care Res*.	Trial	118	2011	Pain caused depression
72	Turk, 1993	*J. Prosthet. Dent*.	Trial	118	1993	Positive correlated
73	Keefe, 1986	*J. Consult Clin. Psych*.	Trial	116	1986	Depression caused pain
74	Braden, 2009	*Gen Hosp. Psychiatr*.	Trial	115	2009	Depression increased use of opioid therapy for non-cancer pain
75	Klauenberg, 2008	*Pain*	Trial	115	2008	Depression reduced pain perception threshold
76	Turner, 2005	*J. Pain*	Trial	114	2005	Self-efficacy for managing pain decreased depression
77	Sharpe, 2001	*J. Psychosom. Res*.	Trial	113	2001	Pain caused depression
78	Chiu, 2005	*Pain*	Trial	113	2005	Depression reduced pain perception threshold
79	Auerbach, 2001	*J. Oral Maxil Surg*.	Trial	112	2001	Pain caused depression
80	Davison, 2005	*J. Pain Symptom. Manage*.	Trial	111	2005	Pain caused depression
81	Gaston, 1999	*Cancer Pract*.	Trial	111	1999	Positive correlated
82	Brown, 2010	*Psycho-Oncology*	Trial	111	2010	Positive correlated
83	MAGNI, 1987	*Pain*	Review	110	1987	Positive correlated
84	Geerlings, 2002	*Soc. Psych. Psych. Epid*.	Trial	107	2002	Positive correlated
85	Coenen, 2011	*Neurosci. Biobehav. R*	Review	107	2011	Positive correlated
86	Jann, 2007	*Pharmacotherapy*	Review	107	2007	Both reduced by antidepressant
87	Munce, 2007	*Psychosomatics*	Trial	105	2007	Pain caused depression
88	Blumer, 1982	*J. Nerv. Ment. Dis*.	Trial	104	1982	Depression caused pain
89	Illi, 2012	*Cytokine*	Trial	103	2012	Positive correlated
90	Haley, 1985	*Pain*	Trial	101	1985	Pain caused depression
91	Kroenke, 2008	*Pain*	Trial	101	2008	Positive correlated
92	Means, 2008	*Depress Anxiety*	Trial	100	2008	Positive correlated
93	Wolfe, 2009	*Arthritis Rheum-Arthr*.	Trial	100	2009	Pain caused depression
94	Kerns, 1988	*J. Consult. Clin. Psych*.	Trial	99	1988	Pain caused depression
95	O'Mahony, 2005	*J. Pain. Symptom. Manage*.	Trial	99	2005	Pain caused depression
96	Foley, 2007	*Am. J. Geriat. Psychiatr*.	Trial	96	2007	Positive correlated
97	Hendeler, 1984	*J. Clin. Psychiatr*.	Trial	96	1984	Pain caused depression
98	Geisser, 2000	*Clin. J. Pain*	Trial	95	2000	Pain caused depression
99	Schwartz, 2014	*Science*	Basic research	95	2014	Pain caused depression
100	Finan, 2013	*Sleep. Med. Rev*.	Review	94	2013	Both related to Dopamin

*Reference details are provided in Supplementary Data Sheet 1*.

## Journals of Top-Cited Studies

Among the 100 top-cited studies, 20 were published in Pain, 6 from Clinical Journal of Pain, 5 from Psychosomatic Medicine, and 4 from Journal of Pain. 3 studies were published in each of the following journals: Jama-Journal of the American Medical Association, Journal of Abnormal Psychology, Journal of Pain and Symptom Management and Journal of Consulting and Clinical Psychology. The other journals had <3 studies. The total citation number of journals ranged from 94 to 3,508. The impact factors of all the journals in 2018 were between 1.438 and 51.273 (Table 2).

**Table 2:** Journals of the 100 top-cited studies published.

**Journal**	**Number of studies**	**Total citation**	**Average citation**	**Impact factor (2018)**
*Pain*	20	3508	175	6.029
*JAMA Intern. Med*.	1	1576	1576	20.768
*Clin. J. Pain*	6	1549	258	2.893
*Psychosom. Med*.	5	1010	202	3.937
*Psychol. Bull*.	2	978	489	16.405
*J. Pain*	4	888	222	2.236
*JAMA J. Am. Med. Assoc*.	3	787	262	51.273
*J. Abnorm. Psychol*.	3	551	184	5.519
*J. Pain. Symptom Manag*.	3	458	153	3.378
*J. Consult. Clin. Psych*.	3	414	138	4.358
*Pediatrics*	2	410	205	5.401
*Arthritis Rheum. Arthr*.	2	370	185	9.002
*J. Gerontol*.	2	285	143	3.418
*Psychosomatics*	2	272	136	1.541
*Brit. J. Psychiatr*.	1	271	271	7.233
*Arthritis Care. Res*.	2	268	134	4.530
*J. Neuroendocrinol*.	1	258	258	3.040
*J. Psychosom. Res*.	2	254	127	2.722
*Psycho-Oncology*	2	243	122	3.430
*J. Clin. Psychiatr*.	2	235	118	4.023
*Nat. Rev. Rheumatol*.	1	222	222	18.545
*Biol. Psychiatr*.	1	219	219	11.501
*Am. J. Psychiatr*.	1	208	208	13.655
*Behav. Res. Ther*.	1	203	203	4.309
*J. Clin. Epidemiol*.	1	196	196	4.650
*Clin. Orthop. Relat. R*	1	189	189	4.154
*Rheumatology*	1	184	184	5.149
*J. Clin. Psychol*.	1	178	178	2.059
*Front. Biosci*.	1	175	175	2.214
*Cancer*	1	164	164	6.102
*J. Clin. Invest*.	1	158	158	12.282
*Pharmacol. Rev*.	1	143	143	18.886
*J. Rheumatol*.	1	141	141	3.634
*Hum. Psychopharm. Clin*.	1	138	138	2.265
*J. Clin. Oncol*.	1	134	134	28.245
*Pain Med*.	1	128	128	2.758
*Oncol. Nurs. Forum*.	1	126	126	1.438
*Arch. Phys. Med. Rehab*.	1	125	125	2.697
*Arthritis Rheum-Us*	1	118	118	4.530
*J. Prosthet. Dent*.	1	118	118	2.787
*Gen. Hosp. Psychiatr*.	1	115	115	3.220
*J. Oral. Maxil. Surg*.	1	112	112	1.781
*Cancer. Pract*.	1	111	111	1.553^*^
*Neurosci. Biobehav. R*	1	107	107	8.002
*Pharmacotherapy*	1	107	107	3.045
*Soc. Psych. Psych. Epid*.	1	107	107	3.152
*J. Nerv. Ment. Dis*.	1	104	104	1.859
*Cytokine*	1	103	103	3.078
*Depress. Anxiety*	1	100	100	4.935
*Am. J. Geriat. Psychiatr*.	1	96	96	3.488
*Science*	1	95	95	41.037
*Sleep. Med. Rev*.	1	94	94	10.517

* *Impact factor of 2004*.

## Publication Years of 100 Top-Cited Studies

The 100 top-cited studies were published between 1979 and 2014. The year 2003 and 2005 were the years with most citation number (*n* = 8), followed by 2008 (*n* = 7), and 2001 (*n* = 6), 2004 (*n* = 6), and 2009 (*n* = 6). Of all the years, the year 2003 contributed the most citation times (*n* = 2,920).

## Study Types of Top-Cited Studies

Among the 100 top-cited studies, 21 studies were reviews, of which 2 were systematic reviews (SR)/meta-analyses; 79 were articles, of which 73 were observational studies, 5 were randomized controlled trials and 1 was basic research.

## Countries of the Top-Cited Studies

According to the country of origin of authors, the top-cited studies were mostly conducted in the USA (*n* = 74), followed by Canada (*n* = 11), and Germany (*n* = 4). China, England, Italy and Netherlands contributed two studies each, while Australia, Denmark and France only contributed 1 study each.

## Institutions Published at least Two of the Top-Cited Studies

In our review, we listed the institutions contributed more than 1 study. Indiana University from the USA produced the most cited studies (*n* = 8), followed by University of Washington (*n* = 7). University of Pittsburgh and University of Michigan both contributed five studies. Johns Hopkins University produced four studies. The rest of the institutions contributed less than four studies. Of the top 16 institutions, 12 institutions were from the USA, and 14 were the university.

## Authors Published at Least Two Papers As First Author or Corresponding Author of the Top-Cited Studies

We also summarized the first authors or corresponding authors who published more than one studies of the 100 top-cited studies. Among the nine first authors and ten corresponding authors, Kroenke, K published the highest number of papers as both the first author (*n* = 4) and corresponding author (*n* = 4).

## Discussion

With the growing awareness of the link between pain and depression, an increasing number of literatures have focused on the interaction between these two conditions. Although literature reviews of the comorbidity of pain and depression have been conducted (Fishbain et al., 1997; Bair et al., 2003; Gagliese et al., 2007), no bibliometrics study to describe the current situation and trend of this field yet, thus we performed the current review to assess it. This study aimed to review the development and progress of the relationship between pain and depression by identifying the top-cited studies in this field.

The 100 top-cited studies were cited from 94 to 1576 times, published in 52 different journals between 1976 and 2014. As it shown that the number of citations is rising as time goes by, of the 100 top-cited articles, 81 were published 10 years ago. Although, the oldest article may not be the most significant research in this field. Probably because the authors tend to cite the articles which were cited in the papers instead of reading and citing the original articles. *Pain*, with 20 of the 100 top-cited studies, received the highest number of citations of 3,508 times. Of the top 5 most cited journals, three journals featuring the spectrum of pain research, which indicated that studies published in professional journals might attract more attention and achieve higher academic value.

Although the studies of pain and depression have been done worldwide, 74% of the top-cited studies were originated from academic institutions in the USA. The most influential institution was the Indiana University, with eight top-cited articles published from 1984 to 2009. The significant influence of the USA may be attributed to its large number of scientific research institutions and the abundant research funds. Also, there may be some bias in this finding. On one hand, consideration of the subtle connection between pain and depression maybe more focused by developed countries than developing countries. On the other hand, researchers from unknown labs in developing countries are less influential in the field who may not have access or resources to publish articles in renowned journals.

Of all the first author and corresponding author, Kroenke, K contributed most of the top-cited articles, and 2 of the four papers were published in JAMA. In addition to the four studies, he also made contributions in other five papers of the top-cited articles. Dr. Kroenke is an internationally respected expert in physical and psychological symptoms, whose principal research interests include pain and depression, and has made a great contribution to this research field. A study concluded a phenomenon that once in control of the commanding heights of their fields, star scientists tend to hold on to their exalted position for a long time, and a burst of published research after the “star” in that field dies (Azoulay et al., 2019). This phenomenon may indicate that big names in science are in a way suppressing younger colleagues work.

The most highly cited articles in pain and depression were in the field of clinical trials, of which 94% were observational study. Pain and depression are the most common physical and psychological symptom-based conditions (Kroenke et al., 2011), respectively, which based on an individual's subjective feelings. Measurement of pain and depression according to the scores of scales. For these reasons, most of the studies were observational studies instead of basic study.

Studies showed that 65% of depressive patients complained about one or more pain, while 5–85% patients with pain reported depression (Bair et al., 2003; Lepine and Briley, 2004; Miller and Cano, 2009). As presented in Table 1, the relationship between pain and depression were investigated in the 100 papers. Forty seven of the 100 top-cited articles indicated that pain caused depression, 23 papers mentioned they were corelated, 9 papers revealed that depression caused pain, while 3 studies found no significant correlation between pain and depression. Five articles investigated the possible common mechanisms of these two symptoms. Three studies found the level of pain and depression were decreased after antidepressants therapy, which pointed out the direction of future research in mechanism and treatment of comorbidity of pain and depression.

In present study, most of the top-cited studies reported the comorbidity of pain and depression and indicated that pain and depression have strong and similar effects on one another (Kroenke et al., 2011). Based on the 100 top-cited articles, the presence of pain negatively affects the recognition and treatment of depression, and depression in patients with pain is similarly associated with more pain complaints and greater impairment (Bair et al., 2003, 2008). Higher prevalence of depression was found in patients with moderate or severe chronic pain compared to patients with mild or no pain (34.1 vs. 18.3%) (Davison and Jhangri, 2005). And a significantly lower Mental Composite Score t-score was found in chronic pain patients with major depressive disorder had than those with minor or no depression (Elliott et al., 2003). Pain and depression are interrelated and interacted. Lin et al. (2003) found benefits of improved depression care extended beyond reduced depressive symptoms and included decreased pain as well as improved functional status and quality of life. Optimized antidepressant therapy followed by a pain self-management program resulted in substantial improvement in depression as well as moderate reductions in pain severity (Kroenke et al., 2009). Cairns et al. (1996) pointed that changes in pain affected depression more than changes in depression affected pain. A 12-month longitudinal analysis in comorbidity of pain and depression also found that change in pain and depression severity was strong predictor of each other (Kroenke et al., 2011).

Pain and depression might share the same biological pathways and neurotransmitters, indicating the same treatment strategy of both concurrently. These mechanisms include direct effects of cytokines on the neuronal environment or indirect effects via downregulation of G protein–coupled receptor kinase 2, activation of the tryptophan-degrading enzyme indoleamine 2,3-dioxygenase that generates neurotropic kynurenine metabolites, increased brain extracellular gluta-mate, and the switch of GABAergic neurotransmission from inhibition to excitation (Romano and Turner, 1985; Bair et al., 2003; Kim et al., 2012). The mesolimbic dopaminergic system (DA) is also shown to associate with both symptoms of pain and depression. Endogenous opioids have been shown to functionally interact with DA, and are directly implicated in pain processing and depression symptoms in regions with heavy DAergic innervation (Finan and Smith, 2013). Among these mechanisms, one possible way was the brain indoleamine 2,3-dioxygenase 1-mediated regulatory mechanism, which has been suggested as a new strategy for the treatment of both conditions (Kim et al., 2012). In addition, studies showed that pain and depression are parallel and independent. In patients with fibromyalgia, neither the extent of depression nor the presence of comorbid major depression modulates the sensory-discriminative aspects of pain processing, as measured by sensory testing or fMRI. The sensory and affective elements were independent of one another and respond differentially to both pharmacologic and non-pharmacologic interventions (Giesecke et al., 2005).

There are several limitations to our review work. Firstly, there may be the missing number of citations since the citation analysis only based on the Web of Science. Some databases, like Google and Scopus, are not included in the statistical collection of cited frequency in Web of Science. Secondly, we searched the database based on the contents of titles, and some studies which did not contain the keywords in their titles may be missed for inclusion. Thirdly, we used total citations as the measurement of impact, but as times goes by, the older the articles are the more citations they may receive. So, the list of top-cited articles may be dominated by some old articles. Fourthly, since the 100 top-cited articles were published before 2015, the results of relationship between pain and depression may not be the latest discovery.

To the best of our knowledge, this study is the first review of top-cited studies in pain and depression. Despite the limitations, we provided evidence of the status and progress in the research field of pain and depression based on the 100 top-cited articles by identifying the contributions made by authors, institutions, and journals. Given the high prevalence of pain and depression, the mechanisms and treatment of the comorbidity of both symptoms remains a key research field.

## Author Contributions

YZ and LD designed the study and analyzed the data. SL, GL, HW, YZ, and LZ drafted of the manuscript. All authors approved the final version of the manuscript.

### Conflict of Interest

The authors declare that the research was conducted in the absence of any commercial or financial relationships that could be construed as a potential conflict of interest.
