# Supplementary material for: The 100 Top-Cited Studies About Pain and Depression
Source: Front Psychol. 2020 Feb 11;10:3072. doi: 10.3389/fpsyg.2019.03072 (PMC7026489; doi:10.3389/fpsyg.2019.03072)
Supplement: Supplementary file 1 [file Data_Sheet_1.PDF]

The reference of the 100 top cited studies

1. Arnow BA, Hunkeler EM, Blasey CM, Lee J, Constantino MJ, Fireman B, et al. Comorbid depression, chronic pain, and disability in primary care. Psychosom Med (2006) 68(2):262-8. Epub 2006/03/24. doi: 10.1097/01.psy.0000204851.15499.fc. PubMed PMID: 16554392.

2. Arnstein P, Caudill M, Mandle CL, Norris A, Beasley R. Self efficacy as a mediator of the relationship between pain intensity, disability and depression in chronic pain patients. Pain (1999) 80(3):483-91. Epub 1999/05/26. doi: 10.1016/s0304-3959(98)00220-6. PubMed PMID: 10342410.

3. Auerbach SM, Laskin DM, Frantsve LM, Orr T. Depression, pain, exposure to stressful life events, and long-term outcomes in temporomandibular disorder patients. J Oral Maxillofac Surg (2001) 59(6):628-33; discussion 34. Epub 2001/06/09. doi: 10.1053/joms.2001.23371. PubMed PMID: 11381383.

4. Bair MJ, Robinson RL, Eckert GJ, Stang PE, Croghan TW, Kroenke K. Impact of pain on depression treatment response in primary care. Psychosom Med (2004) 66(1):17-22. Epub 2004/01/30. doi: 10.1097/01.psy.0000106883.94059.c5. PubMed PMID: 14747633.

5. Bair MJ, Robinson RL, Katon W, Kroenke K. Depression and pain comorbidity: a literature review. Arch Intern Med (2003) 163(20):2433-45. Epub 2003/11/12. doi: 10.1001/archinte.163.20.2433. PubMed PMID: 14609780.

6. Bair MJ, Wu J, Damush TM, Sutherland JM, Kroenke K. Association of depression and anxiety alone and in combination with chronic musculoskeletal pain in primary care patients. Psychosom Med (2008) 70(8):890-7. Epub 2008/09/19. doi: 10.1097/PSY.0b013e318185c510. PubMed PMID: 18799425; PubMed Central PMCID: PMCPMC2902727.

7. Banks SM, Kerns RDJPB. Explaining high rates of depression in chronic pain: A diathesis-stress framework. (1996) 119(1):95-110.

8. Bar KJ, Brehm S, Boettger MK, Boettger S, Wagner G, Sauer H. Pain perception in major depression depends on pain modality. Pain (2005) 117(1-2):97-103. Epub 2005/08/03. doi: 10.1016/j.pain.2005.05.016. PubMed PMID: 16061323.

9. Bigatti SM, Hernandez AM, Cronan TA, Rand KL. Sleep disturbances in fibromyalgia syndrome: relationship to pain and depression. Arthritis Rheum (2008) 59(7):961-7. Epub

2008/06/26. doi: 10.1002/art.23828. PubMed PMID: 18576297; PubMed Central PMCID: PMCPMC3691959.

10. Blackburn-Munro G, Blackburn-Munro RE. Chronic pain, chronic stress and depression: coincidence or consequence? J Neuroendocrinol (2001) 13(12):1009-23. Epub 2001/11/28. PubMed PMID: 11722697.

11. Blumer D, Zorick F, Heilbronn M, Roth T. Biological markers for depression in chronic pain. J Nerv Ment Dis (1982) 170(7):425-8. Epub 1982/07/01. doi: 10.1097/00005053-198207000-00010. PubMed PMID: 7086402.

12. Braden JB, Sullivan MD, Ray GT, Saunders K, Merrill J, Silverberg MJ, et al. Trends in long-term opioid therapy for noncancer pain among persons with a history of depression. Gen Hosp Psychiatry (2009) 31(6):564-70. Epub 2009/11/07. doi: 10.1016/j.genhosppsych.2009.07.003. PubMed PMID: 19892215; PubMed Central PMCID: PMCPMC2774904.

13. Brander V, Gondek S, Martin E, Stulberg SD. Pain and depression influence outcome 5 years after knee replacement surgery. Clin Orthop Relat Res (2007) 464:21-6. Epub 2007/07/03. doi: 10.1097/BLO.0b013e318126c032. PubMed PMID: 17603386.

14. Brown GK. A causal analysis of chronic pain and depression. J Abnorm Psychol (1990) 99(2):127-37. Epub 1990/05/01. doi: 10.1037//0021-843x.99.2.127. PubMed PMID: 2348006.

15. Brown GK, Nicassio PM, Wallston KA. Pain coping strategies and depression in rheumatoid arthritis. J Consult Clin Psychol (1989) 57(5):652-7. Epub 1989/10/01. doi: 10.1037//0022-006x.57.5.652. PubMed PMID: 2794186.

16. Brown LF, Kroenke K, Theobald DE, Wu J, Tu W. The association of depression and anxiety with health-related quality of life in cancer patients with depression and/or pain. Psychooncology (2010) 19(7):734-41. Epub 2009/09/25. doi: 10.1002/pon.1627. PubMed PMID: 19777535; PubMed Central PMCID: PMCPMC2888919.

17. Cairns DM, Adkins RH, Scott MD. Pain and depression in acute traumatic spinal cord injury: origins of chronic problematic pain? Arch Phys Med Rehabil (1996) 77(4):329-35. Epub 1996/04/01. doi: 10.1016/s0003-9993(96)90079-9. PubMed PMID: 8607754.

18. Campbell LC, Clauw DJ, Keefe FJ. Persistent pain and depression: a biopsychosocial perspective. Biol Psychiatry (2003) 54(3):399-409. Epub 2003/08/02. doi: 10.1016/s0006-3223(03)00545-6. PubMed PMID: 12893114.

19. Campo JV, Bridge J, Ehmann M, Altman S, Lucas A, Birmaher B, et al. Recurrent abdominal pain, anxiety, and depression in primary care. Pediatrics (2004) 113(4):817-24. Epub 2004/04/03. doi: 10.1542/peds.113.4.817. PubMed PMID: 15060233.

20. Carroll LJ, Cassidy JD, Cote P. Depression as a risk factor for onset of an episode of troublesome neck and low back pain. Pain (2004) 107(1-2):134-9. Epub 2004/01/13. doi: 10.1016/j.pain.2003.10.009. PubMed PMID: 14715399.

21. Chiu YH, Silman AJ, Macfarlane GJ, Ray D, Gupta A, Dickens C, et al. Poor sleep and depression are independently associated with a reduced pain threshold. Results of a population based study. Pain (2005) 115(3):316-21. Epub 2005/05/25. doi: 10.1016/j.pain.2005.03.009. PubMed PMID: 15911158.

22. Chwastiak LA, Von Korff M. Disability in depression and back pain: evaluation of the World Health Organization Disability Assessment Schedule (WHO DAS II) in a primary care setting. J Clin Epidemiol (2003) 56(6):507-14. Epub 2003/07/23. doi: 10.1016/s0895-4356(03)00051-9. PubMed PMID: 12873644.

23. Ciaramella A, Poli P. Assessment of depression among cancer patients: the role of pain, cancer type and treatment. Psychooncology (2001) 10(2):156-65. Epub 2001/03/27. PubMed PMID: 11268142.

24. Ciechanowski P, Sullivan M, Jensen M, Romano J, Summers H. The relationship of attachment style to depression, catastrophizing and health care utilization in patients with chronic pain. Pain (2003) 104(3):627-37. Epub 2003/08/21. doi: 10.1016/s0304-3959(03)00120-9. PubMed PMID: 12927635.

25. Coenen VA, Schlaepfer TE, Maedler B, Panksepp J. Cross-species affective functions of the medial forebrain bundle-implications for the treatment of affective pain and depression in humans. Neurosci Biobehav Rev (2011) 35(9):1971-81. Epub 2010/12/28. doi: 10.1016/j.neubiorev.2010.12.009. PubMed PMID: 21184778.

26. Currie SR, Wang J. Chronic back pain and major depression in the general Canadian population. Pain (2004) 107(1-2):54-60. Epub 2004/01/13. doi: 10.1016/j.pain.2003.09.015. PubMed PMID: 14715389.

27. Davison SN, Jhangri GS. The impact of chronic pain on depression, sleep, and the desire to withdraw from dialysis in hemodialysis patients. J Pain Symptom Manage (2005) 30(5):465-73. Epub 2005/11/29. doi: 10.1016/j.jpainsymman.2005.05.013. PubMed PMID: 16310620.

28. Dickens C, McGowan L, Dale S. Impact of depression on experimental pain perception: a systematic review of the literature with meta-analysis. Psychosom Med (2003) 65(3):369-75. Epub 2003/05/24. doi: 10.1097/01.psy.0000041622.69462.06. PubMed PMID: 12764209.

29. Diepenmaat AC, van der Wal MF, de Vet HC, Hirasing RA. Neck/shoulder, low back, and arm pain in relation to computer use, physical activity, stress, and depression among Dutch adolescents. Pediatrics (2006) 117(2):412-6. Epub 2006/02/03. doi: 10.1542/peds.2004-2766. PubMed PMID: 16452360.

30. Dworkin RH, Gitlin MJ. Clinical aspects of depression in chronic pain patients. Clin J Pain (1991) 7(2):79-94. Epub 1991/06/01. PubMed PMID: 1809423.

31. Edwards RR, Cahalan C, Mensing G, Smith M, Haythornthwaite JA. Pain, catastrophizing, and depression in the rheumatic diseases. Nat Rev Rheumatol (2011) 7(4):216-24. Epub 2011/02/02. doi: 10.1038/nrrheum.2011.2. PubMed PMID: 21283147.

32. Eisenach JC, Pan PH, Smiley R, Lavand'homme P, Landau R, Houle TT. Severity of acute pain after childbirth, but not type of delivery, predicts persistent pain and postpartum depression. Pain (2008) 140(1):87-94. Epub 2008/09/27. doi: 10.1016/j.pain.2008.07.011. PubMed PMID: 18818022; PubMed Central PMCID: PMCPMC2605246.

33. Elliott TE, Renier CM, Palcher JA. Chronic pain, depression, and quality of life: correlations and predictive value of the SF-36. Pain Med (2003) 4(4):331-9. Epub 2004/01/31. doi: 10.1111/j.1526-4637.2003.03040.x. PubMed PMID: 14750909.

34. Finan PH, Smith MT. The comorbidity of insomnia, chronic pain, and depression: dopamine as a putative mechanism. Sleep Med Rev (2013) 17(3):173-83. Epub 2012/07/04. doi: 10.1016/j.smrv.2012.03.003. PubMed PMID: 22748562; PubMed Central PMCID: PMCPMC3519938.

35. Fishbain DA, Cutler R, Rosomoff HL, Rosomoff RS. Chronic pain-associated depression: antecedent or consequence of chronic pain? A review. Clin J Pain (1997) 13(2):116-37. Epub 1997/06/01. PubMed PMID: 9186019.

36. Foley DJ, Vitiello MV, Bliwise DL, Ancoli-Israel S, Monjan AA, Walsh JK. Frequent napping is associated with excessive daytime sleepiness, depression, pain, and nocturia in older adults: findings from the National Sleep Foundation '2003 Sleep in America' Poll. Am J Geriatr Psychiatry (2007) 15(4):344-50. Epub 2007/03/27. doi: 10.1097/01.Jgp.0000249385.50101.67. PubMed PMID: 17384317.

37. Gaston-Johansson F, Fall-Dickson JM, Bakos AB, Kennedy MJ. Fatigue, pain, and depression in pre-autotransplant breast cancer patients. Cancer Pract (1999) 7(5):240-7. Epub 2000/02/25. PubMed PMID: 10687593.

38. Geerlings SW, Twisk JW, Beekman AT, Deeg DJ, van Tilburg W. Longitudinal relationship between pain and depression in older adults: sex, age and physical disability. Soc Psychiatry Psychiatr Epidemiol (2002) 37(1):23-30. Epub 2002/04/03. PubMed PMID: 11926200.

39. Geisser ME, Robinson ME, Keefe FJ, Weiner ML. Catastrophizing, depression and the sensory, affective and evaluative aspects of chronic pain. Pain (1994) 59(1):79-83. Epub 1994/10/01. doi: 10.1016/0304-3959(94)90050-7. PubMed PMID: 7854806.

40. Geisser ME, Roth RS, Robinson ME. Assessing depression among persons with chronic pain using the Center for Epidemiological Studies-Depression Scale and the Beck Depression Inventory: a comparative analysis. Clin J Pain (1997) 13(2):163-70. Epub 1997/06/01. PubMed PMID: 9186024.

41. Geisser ME, Roth RS, Theisen ME, Robinson ME, Riley JL, 3rd. Negative affect, self-report of depressive symptoms, and clinical depression: relation to the experience of chronic pain. Clin J Pain (2000) 16(2):110-20. Epub 2000/06/28. PubMed PMID: 10870723.

42. Giesecke T, Gracely RH, Williams DA, Geisser ME, Petzke FW, Clauw DJ. The relationship between depression, clinical pain, and experimental pain in a chronic pain cohort. Arthritis Rheum (2005) 52(5):1577-84. Epub 2005/05/10. doi: 10.1002/art.21008. PubMed PMID: 15880832.

43. Gore M, Brandenburg NA, Dukes E, Hoffman DL, Tai KS, Stacey B. Pain severity in diabetic peripheral neuropathy is associated with patient functioning, symptom levels of anxiety and depression, and sleep. J Pain Symptom Manage (2005) 30(4):374-85. Epub 2005/11/01. doi: 10.1016/j.jpainsymman.2005.04.009. PubMed PMID: 16256902.

44. Haley WE, Turner JA, Romano JM. Depression in chronic pain patients: relation to pain, activity, and sex differences. Pain (1985) 23(4):337-43. Epub 1985/12/01. doi: 10.1016/0304-3959(85)90003-x. PubMed PMID: 4088696.

45. Hassett AL, Cone JD, Patella SJ, Sigal LH. The role of catastrophizing in the pain and depression of women with fibromyalgia syndrome. Arthritis Rheum (2000) 43(11):2493-500. Epub 2000/11/18. doi: 10.1002/1529-0131(200011)43:11<2493::Aid-anr17>3.0.Co;2-w. PubMed PMID: 11083273.

46. Hawker GA, Gignac MA, Badley E, Davis AM, French MR, Li Y, et al. A longitudinal study to explain the pain-depression link in older adults with osteoarthritis. Arthritis Care Res (Hoboken) (2011) 63(10):1382-90. Epub 2010/07/28. doi: 10.1002/acr.20298. PubMed PMID: 20662042.

47. Haythornthwaite JA, Sieber WJ, Kerns RD. Depression and the chronic pain experience. Pain (1991) 46(2):177-84. Epub 1991/08/01. doi: 10.1016/0304-3959(91)90073-7. PubMed PMID: 1749640.

48. Hendler N. Depression caused by chronic pain. J Clin Psychiatry (1984) 45(3 Pt 2):30-8. Epub 1984/03/01. PubMed PMID: 6698950.

49. Illi J, Miaskowski C, Cooper B, Levine JD, Dunn L, West C, et al. Association between pro- and anti-inflammatory cytokine genes and a symptom cluster of pain, fatigue, sleep disturbance, and depression. Cytokine (2012) 58(3):437-47. Epub 2012/03/28. doi: 10.1016/j.cyto.2012.02.015. PubMed PMID: 22450224; PubMed Central PMCID: PMCPMC3340525.

50. Jann MW, Slade JH. Antidepressant agents for the treatment of chronic pain and depression. Pharmacotherapy (2007) 27(11):1571-87. Epub 2007/10/30. doi: 10.1592/phco.27.11.1571. PubMed PMID: 17963465.

51. Karp JF, Scott J, Houck P, Reynolds CF, 3rd, Kupfer DJ, Frank E. Pain predicts longer time to remission during treatment of recurrent depression. J Clin Psychiatry (2005) 66(5):591-7. Epub 2005/05/14. doi: 10.4088/jcp.v66n0508. PubMed PMID: 15889945.

52. Kashikar-Zuck S, Goldschneider KR, Powers SW, Vaught MH, Hershey AD. Depression and functional disability in chronic pediatric pain. Clin J Pain (2001) 17(4):341-9. Epub 2002/01/11. PubMed PMID: 11783815.

53. Keefe FJ, Wilkins RH, Cook WA, Jr., Crisson JE, Muhlbaier LH. Depression, pain, and pain behavior. J Consult Clin Psychol (1986) 54(5):665-9. Epub 1986/10/01. PubMed PMID: 2945848.

54. Kelsen DP, Portenoy RK, Thaler HT, Niedzwiecki D, Passik SD, Tao Y, et al. Pain and depression in patients with newly diagnosed pancreas cancer. J Clin Oncol (1995) 13(3):748-55. Epub 1995/03/01. doi: 10.1200/jco.1995.13.3.748. PubMed PMID: 7884435.

55. Kerns RD, Haythornthwaite JA. Depression among chronic pain patients: cognitive-behavioral analysis and effect on rehabilitation outcome. J Consult Clin Psychol (1988) 56(6):870-6. Epub 1988/12/01. doi: 10.1037//0022-006x.56.6.870. PubMed PMID: 3204197.

56. Kim H, Chen L, Lim G, Sung B, Wang S, McCabe MF, et al. Brain indoleamine 2,3-dioxygenase contributes to the comorbidity of pain and depression. J Clin Invest (2012) 122(8):2940-54. Epub 2012/07/04. doi: 10.1172/jci61884. PubMed PMID: 22751107; PubMed Central PMCID: PMCPMC3408737.

57. Klauenberg S, Maier C, Assion HJ, Hoffmann A, Krumova EK, Magerl W, et al. Depression and changed pain perception: hints for a central disinhibition mechanism. Pain (2008) 140(2):332-43. Epub 2008/10/18. doi: 10.1016/j.pain.2008.09.003. PubMed PMID: 18926637.

58. Kroenke K, Bair MJ, Damush TM, Wu J, Hoke S, Sutherland J, et al. Optimized antidepressant therapy and pain self-management in primary care patients with depression and musculoskeletal pain: a randomized controlled trial. Jama (2009) 301(20):2099-110. Epub 2009/05/28. doi: 10.1001/jama.2009.723. PubMed PMID: 19470987; PubMed Central PMCID: PMCPMC2884224.

59. Kroenke K, Shen J, Oxman TE, Williams JW, Jr., Dietrich AJ. Impact of pain on the outcomes of depression treatment: results from the RESPECT trial. Pain (2008) 134(1-2):209-15. Epub 2007/11/21. doi: 10.1016/j.pain.2007.09.021. PubMed PMID: 18022319.

60. Kroenke K, Theobald D, Wu J, Norton K, Morrison G, Carpenter J, et al. Effect of telecare management on pain and depression in patients with cancer: a randomized trial. Jama (2010) 304(2):163-71. Epub 2010/07/16. doi: 10.1001/jama.2010.944. PubMed PMID: 20628129; PubMed Central PMCID: PMCPMC3010214.

61. Kroenke K, Wu J, Bair MJ, Krebs EE, Damush TM, Tu W. Reciprocal relationship between pain and depression: a 12-month longitudinal analysis in primary care. J Pain (2011) 12(9):964-73. Epub 2011/06/18. doi: 10.1016/j.jpain.2011.03.003. PubMed PMID: 21680251; PubMed Central PMCID: PMCPMC3222454.

62. Lautenbacher S, Spernal J, Schreiber W, Krieg JC. Relationship between clinical pain complaints and pain sensitivity in patients with depression and panic disorder. Psychosom Med

(1999) 61(6):822-7. Epub 1999/12/11. doi: 10.1097/00006842-199911000-00015. PubMed PMID: 10593634.

63. Lepine JP, Briley M. The epidemiology of pain in depression. Hum Psychopharmacol (2004) 19 Suppl 1:S3-7. Epub 2004/09/21. doi: 10.1002/hup.618. PubMed PMID: 15378670.

64. Lin EH, Katon W, Von Korff M, Tang L, Williams JW, Jr., Kroenke K, et al. Effect of improving depression care on pain and functional outcomes among older adults with arthritis: a randomized controlled trial. Jama (2003) 290(18):2428-9. Epub 2003/11/13. doi: 10.1001/jama.290.18.2428. PubMed PMID: 14612479.

65. Lindsay PG, Wyckoff M. The depression-pain syndrome and its response to antidepressants. Psychosomatics (1981) 22(7):571-3, 6-7. Epub 1981/07/01. doi: 10.1016/s0033-3182(81)73478-9. PubMed PMID: 7267947.

66. Magni G. On the relationship between chronic pain and depression when there is no organic lesion. Pain (1987) 31(1):1-21. Epub 1987/10/01. doi: 10.1016/0304-3959(87)90002-9. PubMed PMID: 3320879.

67. Maletic V, Raison CL. Neurobiology of depression, fibromyalgia and neuropathic pain. Front Biosci (Landmark Ed) (2009) 14:5291-338. Epub 2009/06/02. PubMed PMID: 19482616.

68. McWilliams LA, Goodwin RD, Cox BJ. Depression and anxiety associated with three pain conditions: results from a nationally representative sample. Pain (2004) 111(1-2):77-83. Epub 2004/08/26. doi: 10.1016/j.pain.2004.06.002. PubMed PMID: 15327811.

69. Means-Christensen AJ, Roy-Byrne PP, Sherbourne CD, Craske MG, Stein MB. Relationships among pain, anxiety, and depression in primary care. Depress Anxiety (2008) 25(7):593-600. Epub 2007/10/13. doi: 10.1002/da.20342. PubMed PMID: 17932958.

70. Miller LR, Cano A. Comorbid chronic pain and depression: who is at risk? J Pain (2009) 10(6):619-27. Epub 2009/04/29. doi: 10.1016/j.jpain.2008.12.007. PubMed PMID: 19398383.

71. Mullen PD, Laville EA, Biddle AK, Lorig K. Efficacy of psychoeducational interventions on pain, depression, and disability in people with arthritis: a meta-analysis. J Rheumatol Suppl (1987) 14 Suppl 15:33-9. Epub 1987/08/01. PubMed PMID: 3656305.

72. Munce SE, Stewart DE. Gender differences in depression and chronic pain conditions in a national epidemiologic survey. Psychosomatics (2007) 48(5):394-9. Epub 2007/09/20. doi: 10.1176/appi.psy.48.5.394. PubMed PMID: 17878497.

73. Nicassio PM, Wallston KA. Longitudinal relationships among pain, sleep problems, and depression in rheumatoid arthritis. J Abnorm Psychol (1992) 101(3):514-20. Epub 1992/08/01. doi: 10.1037//0021-843x.101.3.514. PubMed PMID: 1500608.

74. O'Mahony S, Goulet J, Kornblith A, Abbatiello G, Clarke B, Kless-Siegel S, et al. Desire for hastened death, cancer pain and depression: report of a longitudinal observational study. J Pain Symptom Manage (2005) 29(5):446-57. Epub 2005/05/21. doi: 10.1016/j.jpainsymman.2004.08.010. PubMed PMID: 15904747.

75. Parmelee PA, Katz IR, Lawton MP. The relation of pain to depression among institutionalized aged. J Gerontol (1991) 46(1):P15-21. Epub 1991/01/01. doi: 10.1093/geronj/46.1.p15. PubMed PMID: 1986040.

76. Raskin J, Wiltse CG, Siegal A, Sheikh J, Xu J, Dinkel JJ, et al. Efficacy of duloxetine on cognition, depression, and pain in elderly patients with major depressive disorder: an 8-week, double-blind, placebo-controlled trial. Am J Psychiatry (2007) 164(6):900-9. Epub 2007/06/02. doi: 10.1176/ajp.2007.164.6.900. PubMed PMID: 17541049.

77. Romano JM, Turner JA. Chronic pain and depression: does the evidence support a relationship? Psychol Bull (1985) 97(1):18-34. Epub 1985/01/01. PubMed PMID: 3983297.

78. Rudy TE, Kerns RD, Turk DC. Chronic pain and depression: toward a cognitive-behavioral mediation model. Pain (1988) 35(2):129-40. Epub 1988/11/01. doi: 10.1016/0304-3959(88)90220-5. PubMed PMID: 3237427.

79. Schwartz N, Temkin P, Jurado S, Lim BK, Heifets BD, Polepalli JS, et al. Chronic pain. Decreased motivation during chronic pain requires long-term depression in the nucleus accumbens. Science (2014) 345(6196):535-42. Epub 2014/08/02. doi: 10.1126/science.1253994. PubMed PMID: 25082697; PubMed Central PMCID: PMCPMC4219555.

80. Sharpe L, Sensky T, Allard S. The course of depression in recent onset rheumatoid arthritis: the predictive role of disability, illness perceptions, pain and coping. J Psychosom Res (2001) 51(6):713-9. Epub 2001/12/26. doi: 10.1016/s0022-3999(01)00266-5. PubMed PMID: 11750293.

81. So WK, Marsh G, Ling WM, Leung FY, Lo JC, Yeung M, et al. The symptom cluster of fatigue, pain, anxiety, and depression and the effect on the quality of life of women receiving treatment for breast cancer: a multicenter study. Oncol Nurs Forum (2009) 36(4):E205-14. Epub 2009/07/08. doi: 10.1188/09.Onf.E205-e214. PubMed PMID: 19581224.

82. Spiegel D, Sands S, Koopman C. Pain and depression in patients with cancer. Cancer (1994) 74(9):2570-8. Epub 1994/11/01. doi: 10.1002/1097-0142(19941101)74:9<2570::aid-cncr2820740927>3.0.co;2-3. PubMed PMID: 7923013.

83. Suhr JA. Neuropsychological impairment in fibromyalgia: relation to depression, fatigue, and pain. J Psychosom Res (2003) 55(4):321-9. Epub 2003/09/26. doi: 10.1016/s0022-3999(02)00628-1. PubMed PMID: 14507543.

84. Sullivan MJ, D'Eon JL. Relation between catastrophizing and depression in chronic pain patients. J Abnorm Psychol (1990) 99(3):260-3. Epub 1990/08/01. doi: 10.1037//0021-843x.99.3.260. PubMed PMID: 2145334.

85. Sullivan MJ, Reesor K, Mikail S, Fisher R. The treatment of depression in chronic low back pain: review and recommendations. Pain (1992) 50(1):5-13. Epub 1992/07/01. doi: 10.1016/0304-3959(92)90107-m. PubMed PMID: 1387469.

86. Sullivan MJ, Rodgers WM, Kirsch I. Catastrophizing, depression and expectancies for pain and emotional distress. Pain (2001) 91(1-2):147-54. Epub 2001/03/10. doi: 10.1016/s0304-3959(00)00430-9. PubMed PMID: 11240087.

87. Tsang A, Von Korff M, Lee S, Alonso J, Karam E, Angermeyer MC, et al. Common chronic pain conditions in developed and developing countries: gender and age differences and comorbidity with depression-anxiety disorders. J Pain (2008) 9(10):883-91. Epub 2008/07/08. doi: 10.1016/j.jpain.2008.05.005. PubMed PMID: 18602869.

88. Turk DC, Okifuji A. Detecting depression in chronic pain patients: adequacy of self-reports. Behav Res Ther (1994) 32(1):9-16. Epub 1994/01/01. doi: 10.1016/0005-7967(94)90078-7. PubMed PMID: 8135727.

89. Turk DC, Okifuji A, Scharff L. Chronic pain and depression: role of perceived impact and perceived control in different age cohorts. Pain (1995) 61(1):93-101. Epub 1995/04/01. doi: 10.1016/0304-3959(94)00167-d. PubMed PMID: 7644253.

90. Turk DC, Zaki HS, Rudy TE. Effects of intraoral appliance and biofeedback/stress management alone and in combination in treating pain and depression in patients with temporomandibular disorders. J Prosthet Dent (1993) 70(2):158-64. Epub 1993/08/01. doi: 10.1016/0022-3913(93)90012-d. PubMed PMID: 8371179.

91. Turner JA, Ersek M, Kemp C. Self-efficacy for managing pain is associated with disability, depression, and pain coping among retirement community residents with chronic pain. J Pain (2005) 6(7):471-9. Epub 2005/07/05. doi: 10.1016/j.jpain.2005.02.011. PubMed PMID: 15993826.

92. Turner JA, Romano JM. Self-report screening measures for depression in chronic pain patients. J Clin Psychol (1984) 40(4):909-13. Epub 1984/07/01. doi: 10.1002/1097-4679(198407)40:4<909::aid-jclp2270400407>3.0.co;2-j. PubMed PMID: 6480856.

93. Von Korff M, Le Resche L, Dworkin SF. First onset of common pain symptoms: a prospective study of depression as a risk factor. Pain (1993) 55(2):251-8. Epub 1993/11/01. doi: 10.1016/0304-3959(93)90154-h. PubMed PMID: 8309712.

94. Von Korff M, Simon G. The relationship between pain and depression. Br J Psychiatry Suppl (1996) (30):101-8. Epub 1996/06/01. PubMed PMID: 8864155.

95. Walker AK, Kavelaars A, Heijnen CJ, Dantzer R. Neuroinflammation and comorbidity of pain and depression. Pharmacol Rev (2014) 66(1):80-101. Epub 2013/12/18. doi: 10.1124/pr.113.008144. PubMed PMID: 24335193; PubMed Central PMCID: PMCPMC3880465.

96. Ward NG, Bloom VL, Friedel RO. The effectiveness of tricyclic antidepressants in the treatment of coexisting pain and depression. Pain (1979) 7(3):331-41. Epub 1979/12/01. doi: 10.1016/0304-3959(79)90089-7. PubMed PMID: 530739.

97. Williamson GM, Schulz R. Pain, activity restriction, and symptoms of depression among community-residing elderly adults. J Gerontol (1992) 47(6):P367-72. Epub 1992/11/01. doi: 10.1093/geronj/47.6.p367. PubMed PMID: 1430858.

98. Wilson KG, Eriksson MY, D'Eon JL, Mikail SF, Emery PC. Major depression and insomnia in chronic pain. Clin J Pain (2002) 18(2):77-83. Epub 2002/03/08. PubMed PMID: 11882770.

99. Wolfe F. Determinants of WOMAC function, pain and stiffness scores: evidence for the role of low back pain, symptom counts, fatigue and depression in osteoarthritis, rheumatoid arthritis and fibromyalgia. Rheumatology (Oxford) (1999) 38(4):355-61. Epub 1999/06/23. doi: 10.1093/rheumatology/38.4.355. PubMed PMID: 10378714.

100. Wolfe F, Michaud K. Predicting depression in rheumatoid arthritis: the signal importance of pain extent and fatigue, and comorbidity. Arthritis Rheum (2009) 61(5):667-73. Epub 2009/05/01. doi: 10.1002/art.24428. PubMed PMID: 19404997.
